# Changes in Urination According to the Sound of Running Water Using a Mobile Phone Application

**DOI:** 10.1371/journal.pone.0126798

**Published:** 2015-05-15

**Authors:** Whi-An Kwon, Sung Han Kim, Sohee Kim, Jae Young Joung, Jinsoo Chung, Kang Hyun Lee, Sang-Jin Lee, Ho Kyung Seo

**Affiliations:** 1 Department of Urology, School of Medicine, Institute of Wonkwang Medical Science, Wonkwang University Sanbon Hospital, Gunpo, Gyeonggi-do, Korea; 2 Center for Prostate Cancer, National Cancer Center, Goyang, Korea; 3 Department of Biometric Research, National Cancer Center, Goyang, Korea; 4 Genitourinary Cancer Branch, National Cancer Center, Goyang, Korea; Cedars-Sinai Medical Center, UNITED STATES

## Abstract

**Objective:**

The sound of running water (SRW) has been effectively used for toilet training during toddlerhood. However, the effect of SRW on voiding functions in adult males with lower urinary tract symptoms (LUTS) has not been evaluated. To determine the effect of SRW on urination in male patients with LUTS, multiple voiding parameters of uroflowmetry with postvoid residual urine (PVR) were assessed according to the presence of SRW played by a mobile application.

**Methods:**

Eighteen consecutive male patients with LUTS were prospectively enrolled between March and April 2014. Uroflowmetry with PVR measured by a bladder scan was randomly performed once weekly for two consecutive weeks with and without SRW in a completely sealed room after pre-checked bladder volume was scanned to be more than 150 cc. SRW was played with river water sounds amongst relaxed melodies from a smartphone mobile application.

**Results:**

The mean age of enrolled patients and their mean International Prostate Symptom Score (IPSS) were 58.9 ± 7.7 years (range: 46–70) and 13.1 ± 5.9, respectively. All patients had not been prescribed any medications, including alpha-blockers or anti-muscarinic agents, in the last 3 months. There was a significant increase in mean peak flow rate (PFR) with SRW in comparison to without SRW (15.7 mL/s vs. 12.3 mL/s, respectively, p = 0.0125). However, there were no differences in other uroflowmetric parameters, including PVR.

**Conclusions:**

The study showed that SRW from a mobile phone application may be helpful in facilitating voiding functions by increasing PFR in male LUTS patients.

## Introduction

Although global scientific advancements have brought increased life expectancy and quality of life, the increased number of aged individuals also leads to several issues that detract from quality of life, and such individuals require more health services. Most aged individuals have accompanying medical problems, including urological voiding problems. Reports on the prevalence of lower urinary tract symptoms (LUTS) in men have shown increases with age with or without underlying pathologies.[1–3] One of the most commonly known pathologies among men with LUTS is benign prostatic hypertrophy (BPH). In men with BPH, prostatic size is physiologically increased, causing anatomical bladder outlet obstruction (BOO) with resultant voiding difficulties with LUTS.[4] Another significant etiology for voiding dysfunction with LUTS is an overactive bladder (OAB) associated with impairment of detrusor muscle contractility.[5]

Although the development of many therapeutic approaches, including medical and surgical treatments, can partially resolve voiding problems, a considerable number of LUTS patients still face economic burden of high medical expenses and decreased of quality of life, particularly if they cannot pay for medical expenses for treatment.[6] Patients with LUTS inevitably spend large quantities of money managing their LUTS during their lives, but in doing so, they must either take pills or undergo invasive procedures to improve both LUTS and health-related quality of life.[6–8] For these reasons, the most ideal way to help patients overcome LUTS is thought to be treatment or improvement using people’s inherent capabilities and free-of-charge therapeutic methods for as long as possible.

The sound of running water (SRW) in a sink has effectively been used for toilet training during toddlerhood. Although the molecular mechanism underlying this phenomenon is not fully understood, easy voiding upon listening to SRW involves a complex integration of neurological, muscular, and behavioral mechanisms.[4,5,9] With the idea that using SRW might reflect inherent vesical reflexes and decrease emotional anxiety, people experiencing difficulty with LUTS might achieve positive effects from voiding with comfort and ease, saving money, or being free of taking regular medication.

In the early 1970s, one hospital in New York successfully witnessed the effect of SRW on voiding that made a considerable impact on patients. In their experiment, selected patients were given a tape recorder with headphones playing water sounds for 30 min in order to ease their bathroom experience.[10] With the current development of scientific technology, however, smart phones with diverse applications enable people to play SRW at any time, even listening during micturition. Unfortunately, no studies have investigated the use of SRW using a mobile application on patients with LUTS. Therefore, this study aimed to assess multiple voiding parameters of uroflowmetry (UFM) with postvoid residual urine (PVR) and their changes according to SRW coming from a mobile application, thus determining its efficacy.

## Material and Methods

This study was approved by the local Institutional Review Board of Wonkwang University Sanbon Hospital (IRB No. 7302–201325) and written consents were obtained from every participant. This study was conducted according to the principles expressed in the Declaration of Helsinki.

In a previous study for LUTS patients,[11–13] and based on our EMR data, mean peak flow rate (PFR) varies from 9.2 mL/s to 12 mL/s (SD from 2.6 to 4.1). We decided that a clinically meaningful change in PFR would be approximately 20%. Thus, assuming that mean (± SD) PFR was 10.6 (± 3.0), 18 patients would be required to have 80% power to detect a 20% paired change in PFR with a significance level of 5% using a two-sided paired t-test.

Between March and April 2014, 18 consecutive patients were prospectively enrolled. Participants were those who presented with LUTS in the urology department for the first time. This study excluded any patients older than 80 years, those with hearing impairment, recent history of LUT surgery, trauma, calculi, urethral stricture, infection, any congenital LUT anomaly, any history of malignancy or other neurological or neurodegenerative diseases affecting bladder function, and those patients who refused participation.

All medical histories, including medication, were accumulated from careful interviews with a research doctor (Dr. WA.K) during each visit to the outpatient urological clinics. All patients had LUTS at the time of study onset, and any medication that might influence LUTS was not given during the study period. All patients underwent a detailed pre-planned clinical evaluation for LUTS including careful history documentation, voiding diary, International Prostate Symptom Score (IPSS), physical examination including digital rectal examination, urinalysis, urine cultures, serum creatinine, serum prostate-specific antigen (PSA), and transrectal ultrasonography (TRUS).

UFM with PVR was assessed two times at 1-week intervals with or without SRW. The order of hearing with or without SRW was randomly allocated according to a random table resulting in an equally separated number of nine patients in each group starting with SRW and without SRW. Voiding was performed after the voidable volume was confirmed over 150 cc by a bladder scan prior to UFM in order to ensure consistency. UFM was performed using uroflowmeter (Medtronic Flow Transducers system [DK-2740], Medtronic Inc., USA) in a separate room blocked from outside noise. When the patient was ready to start to void, the patient started the UFM to push the initiating button as having taught by the researcher and the SRW was playing continuously from a mobile phone until the patient finished voiding. SRW was played using river water sound of the iPhone application Relax Melodies (iLBSoft, Ipnos Software Inc., Canada Montreal) during voiding, and a speaker system (Bose SoundLink Bluetooth Mobile speaker II) was used to regenerate real running water sound. PVR was measured by bladder scan (BioCon-500, Medline Industries Inc., USA) after UFM.

After the completion of the information, including the voiding parameters, the PFR, average flow rate (AFR), voided volume, voiding time, and PVR were statistically compared pair-wisely between the with- and without-sound conditions. All the pairwise data are described as mean ± standard deviation or as frequencies with percentages. Paired t-tests were used to compare the changes of the values from UFM with PVR according to SRW. Statistical analyses were performed using STATA 13.1 (STATA Inc., Texas, USA). Results were considered statistically significant if the two-tailed p-value was < 0.05.

## Results

The mean age of the 18 patients was 58.9 ± 7.7 years (range 46.0–70.0) with a mean total IPSS score of 13.1 ± 5.9. Voiding and storage symptom scores of the IPSS were 7.6 ± 3.7 and 5.5 ± 3.2, respectively. Other demographic data are summarized in Table 1.

**Table 1:** Patient demographic and clinical characteristics (N = 18).

Age (years)	58.9 ± 7.7 years (range: 46.0–70.0)
Underlying disease	
Diabetes	3 (16.7%)
Hypertension	2 (11.1%)
Body mass index (kg/m^2^)	24.5 ± 1.8
PSA (ng/mL)	2.3 ± 4.1
Prostate volume (cc)	34.8 ± 12.3
International Prostate Symptom Score	
Voiding symptoms	7.6 ± 3.7
Storage symptoms	5.5 ± 3.2
Nocturia	1.7 ± 1.4
Quality of life	3.1 ± 1.2
Total symptoms	13.1 ± 5.9

PSA: Prostate-specific antigen

**Fig 1:**
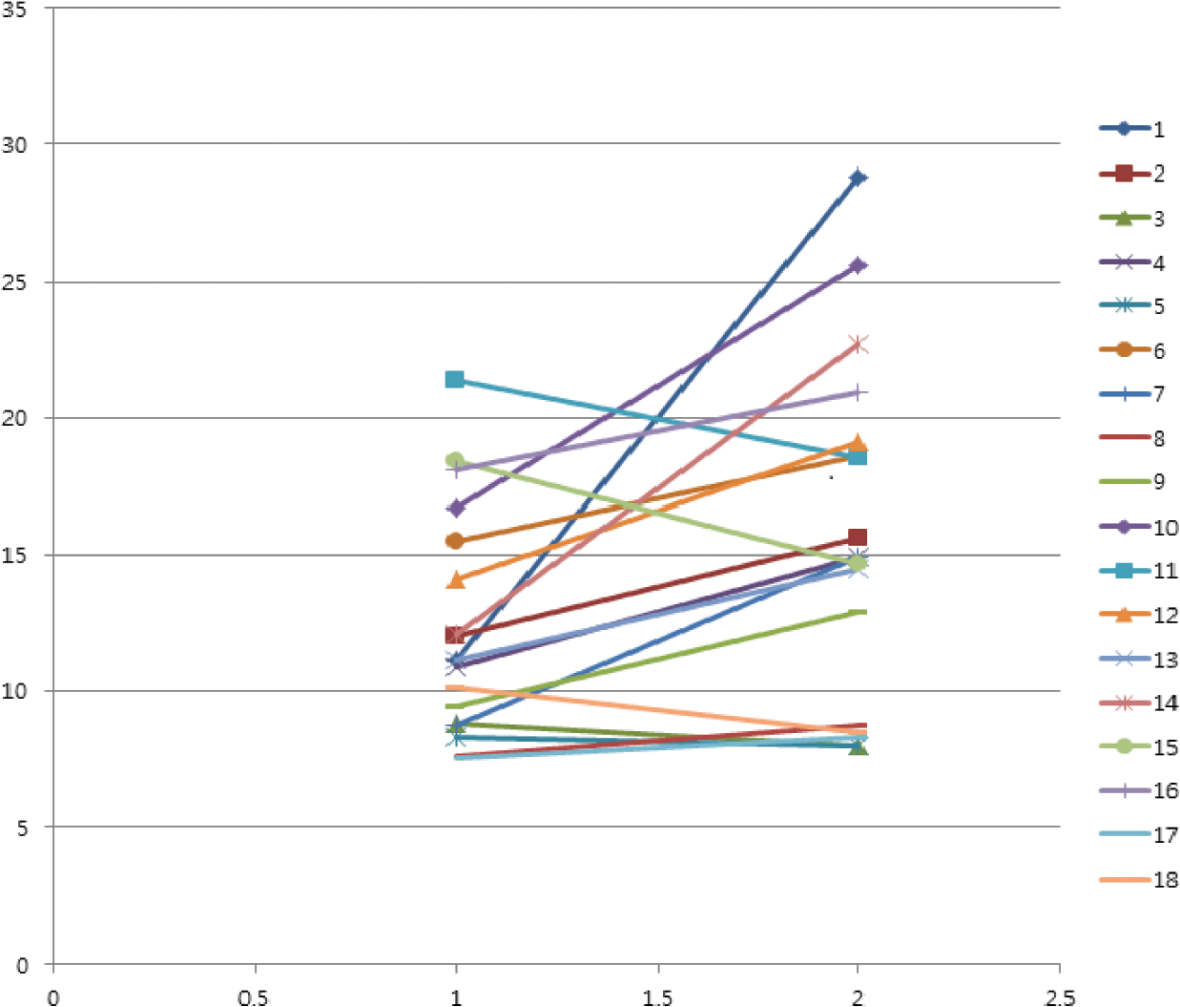
Scatterplot of individuals’ peak flow rates with and without the sound of running water. A significant increase in peak flow rates was observed for the majority of patients following the sound.

For the pairwise voiding parameters of UFM with PVR between with and without listening SRW, PFR with SRW increased in 13 (72%) and decreased in 5 (28%) participants (Fig 1). Mean PFR values with SRW were significantly higher than were those without SRW (15.7 ± 6.2 mL/s vs. 12.3 ± 4.1 mL/s, p = 0.013). However, no differences were observed in other voiding parameters of UFM and PVR, including average flow rate, voided volume, and voiding time (Table 2).

**Table 2:** Changes in urination according to the sound of running water.

Parameter	Without the sound	With the sound	p-value
PFR (mL/s)	12.3 ± 4.1 (10.3–14.4)	15.7 ± 6.2 (12.6–18.8)	0.0125
AFR (mL/s)	7.4 ± 3.5 (5.7–9.2)	8.8 ± 4.38 (6.7–10.9)	0.1555
PVR (mL)	44.8± 35.6 (27.1–62.5)	30.1 ± 20.5 (19.9–40.3)	0.1348
Voiding volume (mL)	232.3 ± 79.4 (192.8–271.8)	274.3 ±120.6 (214.3–334.3)	0.2278
Voiding time (s)	46.2 ± 38.8 (26.9–65.4)	37.7 ± 23.2 (26.1–49.2)	0.2119

Expressed as Mean ± SD

PFR: Peak flow rate

AFR: Average flow rate

PVR: Post-void residual urine volume

Another comparison was performed based on the timing of the sound application (i.e., first or second time listening to SRW). The first attempt at listening to the sound showed a statistically significant increase in PFR, 12.8 ± 4.6 mL/s without SRW against 15.8 ± 6.23 mL/s with SRW (p = 0.023). In the second trial of UFM while listening to SRW, PFR values still increased. However, there were no significant differences in PFR (without SRW, 11.9 ± 3.84 mL/s vs. with SRW, 15.7 ± 7.26 mL/sec; p = 0.131).

## Discussion

To the best of our knowledge, this is the first clinical study to measure changes in voiding parameters as a function of SRW using a mobile phone application. Significantly increased PFR was demonstrated when listening to SRW.

This phenomenon might be explained by the mechanism of parasympathetic acceleration and Pavlovian conditioning, in view of the central and peripheral neural responsive system of the bladder detrusor muscle and urethral sphinter.[14,15] First, SRW might enhance the parasympathetic tone, which powers the detrusor muscle, and relax the resistant tone of the urethral sphincter, thus resulting in the increased PFR. Second, SRW might lead to increased physical and emotional relaxation. Additionally, feeling the urge to urinate with SRW appears to be in line with a conditioned response in Pavlovian conditioning, which was first described in an experiment in which a dog was conditioned to salivate at the sound of a bell.[16] SRW mimics the sound of urination itself as well as feelings of urine passing through the urethra during micturition.

SRW in the sink is a popular method of toilet training in toddlerhood,[17,18] though the underlying mechanism is poorly understood. This phenomenon or circumstance is frequently encountered in urologic clinics when urologists meet patients concerned over urinary incontinence or elderly individuals who experience urgency incontinence, indicating an urge to void or involuntarily urinate after hearing water sounds. Many healthy people feel strong sensations of impulse to void, often resulting in incontinence, after hearing SRW. A previous study reporting on an audio catheter mentioned the practical utility of SRW to stimulate urination.[10]

Nowadays, there is an abundance of mobile phone applications with wearable gear and equipment, relevant to healthcare services, which are aimed at improving quality of life. People are able to replay SRW at any time and place without disturbing others. Many other mobile phone applications have been developed in various medical fields, from diagnosis to therapies, and these have the potential to enlighten the future perspective of medical fields and change the entire medical environment.[19–21] The SRW application fits in this trend as an assistive device for people suffering from voiding problems with a noninvasive, inherent, and inexpensive method.

UFM is valuable for assessing disease severity in men with LUTS, and in particular, PFR is the most representative measure of symptom severity.[22] This study showed that male patients with LUTS can easily use their mobile phones to increase PFR. All patients in this study indicated that they could easily initiate urination with SRW (data not presented). Several meta-analyses and an international randomized controlled trial to evaluate the efficacy of alpha blockers to treat LUTS showed a significant PFR increase of 2.2 mL/s compared with the control group.[13,23–25] This is similar to the present findings, which indicated a mean PFR increase of 3.4 mL/s.

There were some limitations to this study, given the different possible diagnostic disease entities and the short-term follow-up period. To evaluate differences in other voiding parameters of UFM and PVR and differences according to timing of the sound application, the sample size was small. Voiding symptom assessment was not followed with IPSS to evaluate the objective change of voiding symptom into scales. Urodynamic studies were not applied to evaluate baseline bladder function and sphincteric function of the urethra. In addition, this study did not evaluate every possible water sound mobile phone application to determine the most effective and adequate sound resonance and wavelength of SRW. Further, other subjective psychological factors were not evaluated. However, the efficacy of hearing SRW showed its effects in the PFR increase the described comfort with initiating voiding. Thus, further studies that compensate for these limitations are warranted, and it is expected that similarly favorable outcomes would be obtained for helping patients with voiding problems in various ways, especially for those patients with paruresis who might be expected beneficiary and effective for this stimulant and behavioral modification treatment patients with LUTS including hesitancy who might more easily initiate urination with a higher PFR. In future studies, a specific SRW with a comparative control group is necessary to determine the factors associated with the basic functional mechanism by which SRW helps to facilitate urination and the appropriate frequency of SRW for helping patients with voiding problems among various sounds.

## Conclusions

A significant increase in PFR was demonstrated when patients heard SRW using a mobile phone application. Based on these results, it appears that SRW by sound application can help male patients with LUTS improve voiding problems and maintain a normal lifestyle.
